# Copy Number Variation and SNP Affect Egg Production in Chickens by Regulating AP2M1 Expression to Inhibit GnRH Synthesis

**DOI:** 10.3390/ani15202990

**Published:** 2025-10-15

**Authors:** Dandan Wang, Yanchun Yu, Yiqian Zhu, Cancan Liu, Qiuhong Fu, Baoguo Liu, Yongqiang Wang, Jiyuan Shen, Guanghui Wei, Xiaojun Liu

**Affiliations:** 1 College of Animal Science and Veterinary Medicine, Henan Institute of Science and Technology, Xinxiang 453003, China; ddwang2023@hist.edu.cn (D.W.); 13938734169@stu.hist.edu.cn (Y.Y.); 18530209395@stu.hist.edu.cn (Y.Z.); 18336553502@stu.hist.edu.cn (C.L.); fqh@stu.hist.edu.cn (Q.F.); liubaoguo@hist.edu.cn (B.L.); wangyongqiang@hist.edu.cn (Y.W.); shenjy@hist.edu.cn (J.S.); 2 College of Animal Science and Technology, Henan Agricultural University, Zhengzhou 450046, China

**Keywords:** chicken, egg production, AP2M1, GnRH regulation, copy number variation (CNV), single nucleotide polymorphism (SNP)

## Abstract

**Simple Summary:**

Egg production trait has always been a key focus in the selection of chicken laying-oriented breeding, which is a complex trait regulated by multiple genes, and therefore, excavating egg laying-related key genes and genetic markers will contribute to molecular design breeding. Here, we conducted the tissue expression characteristic analysis of *AP2M1* in vivo and its functional gain and loss tests in vitro and verified that the negative impact of hypothalamic *AP2M1* expression on chicken egg production is achieved by inhibiting the synthesis and secretion of GnRH hormone in hypothalamic neurons. Furthermore, we identify egg laying-related functional variations including a CNV containing *AP2M1* genomic sequence and an intronic SNP chr9:15994879T>C in *AP2M1*. These functional variations have enriched the genetic basis of egg production trait and will facilitate the molecular breeding improvement of these traits.

**Abstract:**

Deciphering egg laying-related genetic basis and aggregating its key genes or genetic markers will be helpful for genetic improvement of chicken laying-oriented breeding. Our previous research found adaptor related protein complex 2 mu 1 subunit (*AP2M1*) gene is a key candidate gene related to egg production. However, its functions and genetic regulatory mechanisms remain unclear. This study aims to clarify *AP2M1* functions and identify its functional variants. Expression characteristic analysis of *AP2M1* within and between breeds confirmed the negative regulatory relationship of hypothalamic *AP2M1* expression on egg production. Overexpression and interference tests indicated that *AP2M1* inhibited GnRH synthesis and secretion in chicken hypothalamic neuron cells. To explore molecular markers influencing *AP2M1* expression, a copy number variation (CNV) region containing *AP2M1* were verified in different chicken breeds by qRT-PCR; a copy number loss of *AP2M1* were observed in layers compared to native breeds, commercial broilers, and wild breed. Correlation analysis between CNV and egg number, as well as differential expression analysis of different copy numbers, indicated that the CNV contributed to the differences in egg production by influencing *AP2M1* expression. Meanwhile, through association analysis of whole-genome SNPs in *AP2M1* with 13 egg production traits, 15 egg-laying related SNPs were identified. Further difference expression analysis among the different genotypes of SNPs and dual-luciferase reporter assay confirmed that chr9:15994879T>C was a functional SNP regulating *AP2M1* expression. These findings unveil egg laying-related functional molecular markers will help accelerate molecular design breeding process of chicken egg production.

## 1. Introduction

Egg production trait is one of the most core economic traits in poultry production and thus has always been a key focus in the selection of chicken laying-oriented breeding. Due to the fact that the egg production trait is a complex trait regulated by multiple micro-effect genes, the selection accuracy via traditional breeding strategies is greatly limited [1]. Deciphering the genetic basis of egg production trait and aggregating its key dominant genes or genetic markers will be conducive to the genetic improvement of chicken laying-oriented breeding.

Native chicken breeds possess remarkable advantages such as excellent meat and egg quality, as well as strong adaptability and disease resistance. They are precious resources for breeding new varieties. However, native chicken breeds generally have the drawback of low egg production. For instance, Lushi chicken and Gushi chicken, which are native breeds in China, have an average annual egg production of only 165–190 eggs, which is 130 to 150 eggs less than that of high-yield layer breeds such as Lohmann and Isa (NLPGRC 2011). These have restricted the development of native chicken industry. Therefore, the genetic improvement of the egg-laying performance of native chicken breeds is urgently needed.

Our previous study demonstrated that the expression of the adaptor-related protein complex 2 mu 1 subunit (*AP2M1*) gene in the hypothalamus was significantly higher during the pre-laying period (15 weeks of age) than during the peak-laying period (30 weeks of age) in Lushi chickens. Furthermore, in Gushi chickens, hypothalamic *AP2M1* mRNA levels were significantly negatively correlated with both total egg production and serum concentrations of FSH, LH, and progesterone [2]. These results suggest *AP2M1* is a key candidate gene for the negative regulation of egg production, yet its specific regulatory role in chickens awaits functional characterization. In mammals, *AP2M1* gene, encoding μ-subunit of adaptor protein complex 2 (AP-2), links clathrin to the endocytic membrane to play a role in clathrin-mediated endocytosis (CME) and synaptic vesicle recycling [3,4]. Synaptic vesicle recycling is the basis of neurotransmitter release, which is essential for information transmission between neurons in the nervous system [5]. Recent research has shown that an abnormal AP2M1 phosphorylation cycle can trigger endocytic defects and dopaminergic neurodegeneration, which are implicated in Parkinson’s disease risk [6]. Furthermore, a direct impact of *AP2M1* loss on CME has been demonstrated by findings that its knockout in mouse astrocytes significantly impairs the endocytosis of transferrin [7]. Given its critical role in synaptic function and neurotransmitter release, we hypothesized that *AP2M1* could also regulate the secretion of key neurohormones, such as GnRH, which governs reproduction.

Further, at the genomic level, it remains unexplored which genetic markers will affect the expression regulation of the *AP2M1* gene in chickens. We know that domestication and artificial selection alter the genome variation characteristics and thus drive the phenotypic changes among different breeds or lines [8]. Genomic genetic variation types mainly include single nucleotide polymorphisms (SNPs), insertion or deletion of short segments (InDels), and genomic structural variations [9]. Copy number variations (CNVs) are a form of genomic structural variations, ranging from approximately 1 Kb to 3 Mb in size [10], which can affect gene expression levels through containing or disrupting multiple gene coding regions or regulatory elements [11]. Currently, a large number of candidate genes or genome variation markers that potentially affect the economically important traits or appearance traits have been identified by different genetic analysis approaches in various chicken populations [12]. For instance, a CNV located in the exon 1 of *TMEM86A* was significantly associated with chicken egg yolk weight and eggshell strength [13]. Additionally, a SNP chr3:79510218A>T located on the intron 3 of *PHIP* affected egg production by altering *PHIP* expression in chickens [14]. For the *AP2M1* gene in chicken, Yi et al. (2014) identified a copy number gain of *AP2M1* (*n* = 3.0) in a meat-type chicken (Cornish) compared to Chinese indigenous chicken breeds [15,16]. In addition, the genetic association study of egg production traits in Gushi chickens and the multi-tissue transcriptome integration analysis of high- and low-yield groups identified *AP2M1* as one of the egg laying-related core candidate genes [17]. These preliminary studies have provided a direction for the exploration of the potential genetic regulatory mechanisms of *AP2M1*.

In the present study, we first systematically carried out the tissue expression characteristic analysis of *AP2M1* within and between varieties to verify the relationship between *AP2M1* expression and chicken egg production. Subsequently, we investigated the regulatory function of *AP2M1* by overexpression and interference tests in chicken primary hypothalamic neuron cells. Further on, we excavated the CNV and SNP functional variants that contribute to the differences in egg production by influencing the expression level of *AP2M1* in chicken. Our findings lay a theoretical foundation for the development of effective genetic molecular markers for egg production and will accelerate genetic selection in chicken laying-oriented breeding.

## 2. Materials and Methods

### 2.1. Animals and Sample Collection

Two dual-purpose egg and meat-type native breeds from Henan Province of China, namely Gushi (GS) chicken and Lushi (LS) chicken, as well as the high-yield layer breed Hy-Line (HL) chicken, were used for the tissue expression characteristic analysis of *AP2M1*. The female birds of GS and LS chicken were raised in single cages with the same environmental conditions and the recommended feeding procedure after 12 weeks of age. HL chickens were reared with the phased standard commercial diet of Hy-Line Brown (www.hyline.com (accessed on 12 May 2024)).

Three healthy hens of 43-week-old GS chicken were randomly selected and slaughtered humanely. Fourteen types of tissues, including hypothalamus, pituitary, cerebrum, shell gland, lung, abdominal fat, liver, ovary, kidney, duodenum, proventriculus, heart, pancreas, and pectoralis, were collected for the tissue expression profile analysis of *AP2M1*.

Hypothalamus tissues from 20-, 28-, 36-, 43-, and 50-week-old high- (*n* = 6) and low-yield GS chickens (*n* = 6) in the 14th generation with complete individual egg-laying records were collected for the expression difference analysis of *AP2M1* within the breed. This study phase involved twelve 43-week-old high- and low-yield GS hens, which did not overlap with the three hens previously used for tissue expression profile analysis of *AP2M1*. Using individual egg-laying records, we defined high-yield and low-yield groups from 764 GS chickens [17]. We assigned the top 10% of population with egg number as the high-yield group, and the bottom 20% of population as the low-yield group at 20, 28, 36, 43, and 50 weeks of age, respectively.

Hypothalamus tissues of 30-week-old GS chicken (*n* = 6), LS chicken (*n* = 6), and HL chicken (*n* = 6) were collected for the expression difference analysis of *AP2M1* among different breeds. All birds were euthanized at the corresponding weeks of age, and all tissue samples were isolated, immediately snap-frozen in liquid nitrogen, and stored at −80 °C until use.

Sixty-three individuals from ten different breeds were selected for the intervarietal CNV validation of *AP2M1* gene. Ten breeds include the following layer breeds: White Leghorn (WL, *n* = 6), Rhode Island Red (RIR, *n* = 6), and Rhode Island White (RIW, *n* = 6) chickens; native breeds: Silky (SK, *n* = 6), Xichuan black bone (XC, *n* = 6), Houdan (HD, *n* = 6), Lushi (LS, *n* = 6) chickens; broiler breeds: Arbor Acres broilers (AA, *n* = 6) and Cobb broilers (Cobb, *n* = 6); wild breed: Red Jungle Fowl (RJF, *n* = 9). Blood samples from 63 individuals were collected by wing vein for genomic DNA extraction.

### 2.2. Plasmid Construction and siRNA Synthesis

The coding sequence of chicken *AP2M1* gene was amplified from chicken hypothalamic tissue and cloned into pcDNA3.1-EGFP plasmid vector (pcDNA3.1-AP2M1) by using ClonExpress^®^ II One Step Cloning Kit (Vazyme, Nanjing, China) with the restriction enzymes NheI and HindIII. The adjacent 100 nucleotides of chr9:15994879T>C was amplified from chicken genomic DNA and cloned into the pGL3-promoter plasmid vector (Promega, Madison, WI, USA) to construct luciferase reporter plasmid vectors. The primers are listed in Table S1. The interfering fragment of *AP2M1* (si-AP2M1) and its negative control (si-NC) were synthesized by Tsingke Biotech (Tianjin, China).

### 2.3. Chicken Primary Hypothalamic Neuron Cell Isolation, Culture, and Transfection

The hypothalamuses of 17-day-old Hyland Brown chicken embryos were used to isolate primary hypothalamic neuron cells according to our previous protocol [17]. In brief, the hypothalamus was quickly removed and washed in the ice-cold dissecting liquid 1 (98% D-Hanks + 2% penicillin/streptomycin) and dissecting liquid 2 (98% high-glucose DMEM + 2% penicillin/streptomycin). The cell suspension digested with 1 mg/mL collagenase type II (Solarbio, Beijing, China) was filtered using 200-, 500-mesh sieves, respectively. The filtered cell suspension was re-suspended by high-glucose DMEM supplemented with 15% FBS, 1% L-glutamine (Solarbio, Beijing, China), and 1% penicillin/streptomycin to obtain dispersed cells. The cells were then cultured in a 5% polylysine (Sigma, St. Louis, MO, USA)-coated 12-well plate (NUNC) at 37 °C with 5% CO_2_. The medium was replaced by the complete Neurobasal medium (Gibco, Gaithersburg, MD, USA) supplemented with 1% L-glutamine, 2% B27 (Gibco, Gaithersburg, MD, USA), and 1% penicillin/streptomycin, and an additional 5% β-D-cytarabine (Solarbio, Beijing, China) after 36 h. The cells were incubated for 24 h, after which the medium was replaced with complete Neurobasal medium.

Overexpression (pcDNA3.1-AP2M1 and pcDNA3.1-EGFP) and interference (si-AP2M1 and si-NC) experiments were performed using the transfection reagent Lipofectamine 3000 (Invitrogen, Carlsbad, CA, USA) following the manufacturer’s instruction.

### 2.4. Western Blot and Immunofluorescence

The hypothalamic tissues of 28-week-old high- (*n* = 4) and low-yield GS chickens (*n* = 4) were extracted proteins to analyze the protein expression difference in AP2M1. Chicken primary hypothalamic neuron cells of overexpression and interference experiments of *AP2M1* were extracted proteins to analyze whether the overexpression and interference of *AP2M1* at the protein level were successful. The protein samples were incubated with AP50 polyclonal antibody (1:500, 27355-1-AP, Proteintech, Wuhan, China) and mouse anti-GAPDH (1:50,000, 60004-1-Ig, Proteintech, Wuhan, China) antibodies at 4 °C overnight and then incubated with HRP-conjugated goat anti-rabbit (1:50,000, AB-228341, SeraCare, Invitrogen, Carlsbad, CA, USA) for 50 min. The gray values of the blot signals were detected with an AlphaEaseFC 6.0 beta software system (Alpha Innotech Corporation, San Leandro, CA, USA). The GAPDH was used to normalize AP2M1 protein level for each sample.

The cell slides were produced using a 6-well plate with internal coverslips coated with 5% polylysine. Rabbit anti-GnRH (1:1000, PAA843Ga01, Cloud-clone, Wuhan, China) and DYKDDDDK tag recombinant antibody (1:500, 80801-2-RR, proteintech, Wuhan, China) were used to confirm the co-localization relationship between AP2M1^3×flag^ and GnRH on the hypothalamic neuron cell by double-labeling immunofluorescence. The immunofluorescence images were captured by using fluorescence microscope (Olympus, Melville, NY, USA).

### 2.5. Real-Time Quantitative PCR

The gene expression changes or CNVs were detected using real-time quantitative PCR (qRT-PCR). For the gene expression detective, total RNA of tissues and hypothalamic neuron cells were extracted with TRIzol reagent (Invitrogen, Carlsbad, CA, USA), and 1 μg of total RNA per reaction was used to generate the first-strand cDNA using a PrimeScript RT Reagent Kit with gDNA Eraser (Takara, Tokyo, Japan). qRT-PCR amplification was performed with 2×SYBR Premix Ex TaqTM II (Takara, Tokyo, Japan). The 2^−ΔΔCt^ method was used to quantify the relative mRNA level of gene using GAPDH as the internal control gene.

For CNV validation of *AP2M1*, qRT-PCR amplification template used DNA samples from 63 different chicken breeds mentioned above. Each DNA sample was diluted to 10 ng/μL, and the concentrations were verified using a spectrophotometer. The absolute copy numbers of the unknown samples were estimated using a 6-point standard curve method (2.5, 5, 10, 20, 40, and 80 ng/μL) from known sample concentration and ploidy. The *PCCA* gene, identified as a stable, autosomal non-CNV locus with two copies, was used as the control region. The absolute copy numbers were assigned after comparing the cycle threshold (Ct) values with the standard curve and the amount of copies in 1 ng of reference DNA (assumed as one unit) [16]. The absolute copy number rounding principle is as follows: 0.8–1.2 represents one copy (CN = 1), 1.6–2.4 represents 2 copies (CN = 2), 2.6–3.4 represents 3 copies (CN = 3), 3.6–4.4 represents 4 copies (CN = 4), and 4.6–5.4 represents 5 copies (CN = 5).

All specific primers used in this study were designed using NCBI Primer-BLAST tool and synthesized at Tsingke Biotechnology Co., Ltd. (Beijing, China) (Table S1). Each sample was tested in triplicate.

### 2.6. Enzyme-Linked Immunosorbent Assay for GnRH

The supernatant of hypothalamic neuron cells treated with *AP2M1* overexpression and interference were collected to measure GnRH hormone levels by using a chicken GnRH ELISA Kit (Jiangsu Meimian industrial Inc., Yancheng, China) according to the manufacturer’s instruction. Three technological duplications were performed for each serum sample.

### 2.7. SNP Characteristics Mapping to Chicken AP2M1 Gene

SNPs mapping to the 2 kb promoter region and gene body of *AP2M1* were extracted from our previous whole-genome resequencing data of 75 chickens from 3 different egg-laying types, including 20 layers (WL and RIR), 50 native chickens (GS, LS, XCBB, ZYSH, and HNG), and 5 wild ancestors (RJF) [17,18]. The detailed information on genomic SNPs in *AP2M1* is available in Table S2. Genetic variation patterns of SNPs in *AP2M1* gene were identified in the above multiple chicken breeds by PCA.

Association study of genomic SNPs in *AP2M1* with egg production traits, including egg number (EN) at different laying periods (EN21-25w, EN26-30w, EN31-35w, EN36-43w, EN31-43w, and ENT), average clutch size (ACS) at different laying periods (ACS21-25w, ACS26-30w, ACS31-35w, ACS36-43w, ACS31-43w, and ACST), and maximum clutch size (MCS) in the 12 generations of 900 GS chickens were carried out based on SNP-based GWAS [17]. Briefly, association analyses were performed using the mixed linear model (MLM) in the GCTA v1.92.265. The population stratification was controlled by adding the first three PCs as covariates, and the batch effect was controlled by adding the birth batch as a covariable. The statistical model was:

*y* = *Wa* + *Xb* + *u* + *e*

where *y* is the vector of phenotype; *W* is an matrix of covariates (fixed effects contain first three PCs, birth batch and a column of 1 s); *a* is a vector of the corresponding coefficients including the intercept; *X* is an vector of SNP genotypes; *b* is the effect size of the SNP; *u* is vector of individual random effects; *e* is vector of errors. SNPs were defined as an egg laying-related small effect SNP with a *p* < 1 × 10^−4^.

Egg laying-related SNPs in *AP2M1* underwent PCA across the multiple aforementioned breeds. LD analysis was subsequently carried out via Haploview 4.2.

The differential expression analysis of *AP2M1* among the different genotypes of 5 SNPs including chr9:15980929G>A, chr9:15987945T>A, chr9:15993362G>A, chr9:15994879T>C, and chr9:15995354G>A were performed in 53 individuals of the 14th generation GS chicken population. The SNP genotypes were detected by using Kompetitive Allele Specific PCR (KASP) technique [19]. The association analysis between SNP genotypes and *AP2M1* expression in hypothalamus of 43-week-old GS chickens were performed by mixed linear model.

### 2.8. Dual-Luciferase Reporter Assay

A total of 0.9 μg of the constructed luciferase reporter plasmid vectors and 0.1 μg of pRL-TK Renilla luciferase vector (Promega, Madison, WI, USA) were transfected into chicken primary hypothalamic neuron cells by using transfection reagent Lipofectamine 3000 (Invitrogen, Carlsbad, CA, USA). Dual-Luciferase Reporter Assay System (Promega, Madison, WI, USA) was used to detect cell luciferase activity. Fireflyluciferase activity was normalized to Renilla luciferase.

### 2.9. Statistical Analysis

Statistical analysis was performed using IBM SPSS 23.0 (IBM, Chicago, IL, USA). The association between SNPs and *AP2M1* expression was determined using the linear mixed model. The model used was as follows:

Y*_j__lm_* = μ + G*_j_* + f*_l_* + e*_j__lm_*

In the model, Y*_jlm_* was the individual *AP2M1* expression value, µ was the observation mean, G*_j_* was the fixed effect of genotype, f*_l_* is the random effect of the family, and e*_j__lm_* is the random error. The influence of the different genotypes on *AP2M1* expression was investigated by least squares analysis [20].

*p* < 0.05 was considered statistically significant, and Bonferroni’s test was used as control for multiple comparisons.

Statistical significance between the two groups was calculated by an independent samples *t*-test. The results were presented as the mean ± SEM. * *p* < 0.05, ** *p* < 0.01, and *** *p* < 0.001.

## 3. Results

### 3.1. Expression Level of AP2M1 Gene in Hypothalamus Is Negatively Correlated with Chicken Egg Production

**Figure 1:**
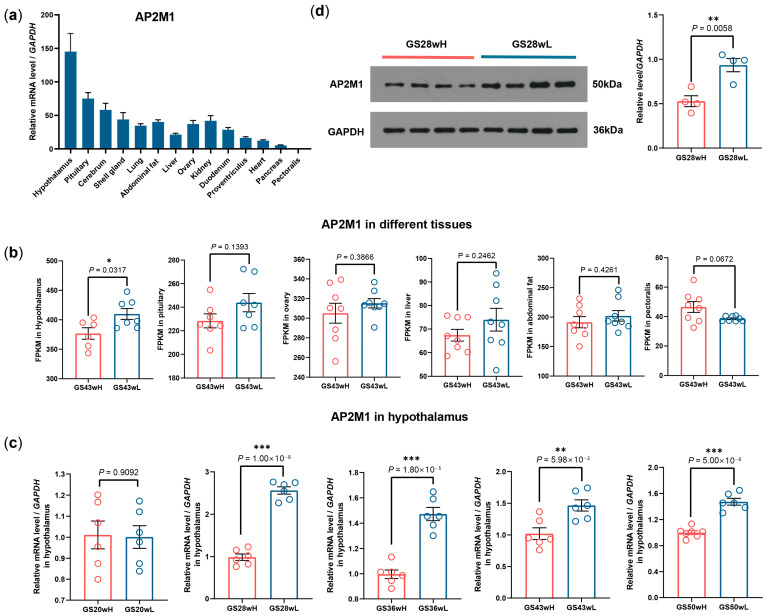
Expression characteristics of *AP2M1* gene in chicken. (**a**) Tissue expression profiles of *AP2M1* in 43-week-old Gushi (GS) chickens (*n* = 3). (**b**) FPKM of *AP2M1* in different tissues between 43-week-old high- (GS43wH) and low-yield GS chickens (GS43wL) (*n* = 6 for hypothalamus, *n* = 7 for pituitary, *n* = 8 for ovary, liver, abdominal fat, and pectoralis). (**c**) The mRNA expression difference in *AP2M1* in hypothalamus between high- and low-yield GS chickens at different egg-laying stages (*n* = 6). (**d**) The protein expression difference in *AP2M1* in 28-week-old high- and low-yield GS chickens (*n* = 4). The data are presented as mean ± SEM. Signifcant diference: * *p* < 0.05, ** *p* < 0.01, *** *p* < 0.001.

The mRNA expression level of chicken *AP2M1* was first detected in different tissues in 43-week-old GS chickens. *AP2M1* gene exhibited a widespread expression pattern across multiple tissues, but its expression was particularly high in the hypothalamus and pituitary (Figure 1a). Using the transcriptome data of 6 tissues (hypothalamus, pituitary gland, ovary, liver, abdominal fat, and pectoral muscle) from the 43-week-old high- and low-yield groups of GS chickens that we previously constructed [17], we conducted a differential expression analysis of the *AP2M1* gene. The result showed that *AP2M1* exhibited significant differential expressions in the hypothalamus and not in the other five tissues, and its expression level in the high-yield group (GS43wH) was significantly lower than that in the low-yield group (GS43wL). (*p* < 0.05; Figure 1b). Subsequently, the significant negative correlation was confirmed between the *AP2M1* mRNA levels in the hypothalamus of GS chickens and their egg production at 28, 36, 43, and 50 weeks of age (*p* < 0.01; Figure 1c). The expression characteristics of hypothalamus AP2M1 protein in the high- and low-yield groups were consistent with those of its mRNA (*p* < 0.01; Figure 1d).

**Figure 2:**
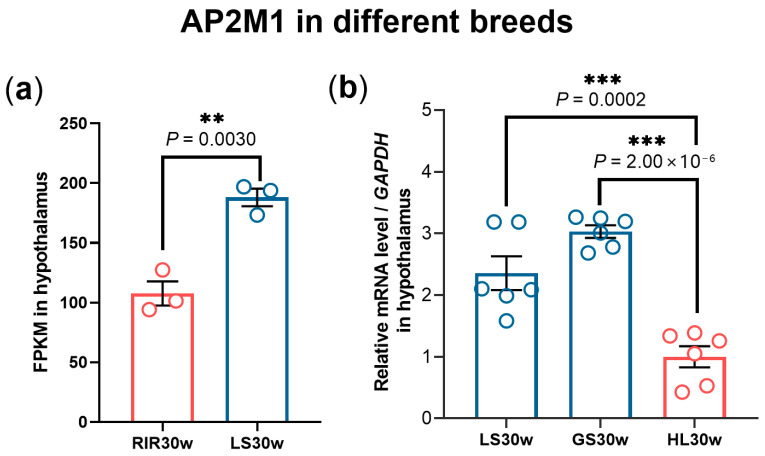
Expression pattern of hypothalamus *AP2M1* in different breeds. (**a**) FPKM of hypothalamus *AP2M1* in 30-week-old Rhode Island Red chickens (RIR30w) and Lushi chickens (LS30w) (n = 3). (**b**) The mRNA level of hypothalamus *AP2M1* in 30-week-old. Hy-line layers (HL30w), Gushi chickens (GS30w), and LS30w (*n* = 6). The data are presented as mean ± SEM. Significant difference: ** *p* < 0.01, *** *p* < 0.001.

Further, we investigated the expression pattern of hypothalamus *AP2M1* in different breeds and found that the expression level of *AP2M1* was significantly lower in high-yield layers (RIR and HL chickens) compared to native chicken breeds (LS and GS chickens) (*p* < 0.01; Figure 2). These results confirm that *AP2M1* negatively affects chicken egg production among different breeds or within the same breed.

### 3.2. AP2M1 Inhibits GnRH Synthesis and Secretion in Chicken Hypothalamic Neuron Cells

**Figure 3:**
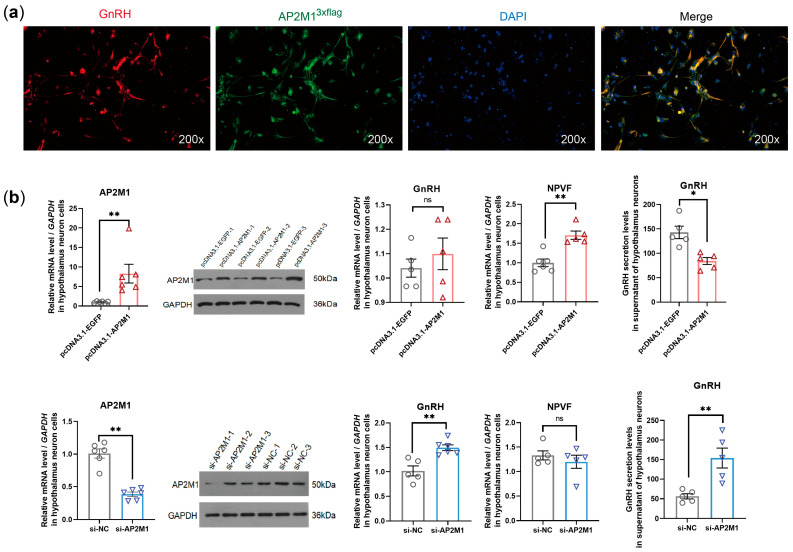
Functional validation of *AP2M1* in chicken primary hypothalamic neuron cells. (**a**) Immunofluorescence co-localization detection of AP2M1 and GnRH in chicken primary hypothalamic neuron cells. (**b**) Effects of *AP2M1* overexpression or interference on reproductive hormone GnRH expression and secretion (*n* = 5–6 for each group in the mRNA level and secretion level, *n* = 3 for each group in the protein level). The data are presented as mean ± SEM. “ns” indicates no significant difference. Signifcant diference: * *p* < 0.05, ** *p* < 0.01.

To investigate the regulatory function of *AP2M1* in chicken primary hypothalamic neuron cells. We first confirmed the co-localization relationship between AP2M1^3×flag^ and GnRH on the hypothalamic neuron by double-labeling immunofluorescence (Figure 3a). Overexpression and interference tests of *AP2M1* indicated that the overexpression of *AP2M1* upregulated the expression level of neuropeptide VF precursor (NPVF, also named GnIH, a gonadotropin-inhibiting hormone) and decreased the secretion level of GnRH hormone, while the interference of *AP2M1* promoted the expression and secretion of GnRH (*p* < 0.05; Figure 3b), suggesting that *AP2M1* exerts a negative regulatory effect on the synthesis and secretion of the hypothalamic reproductive hormone GnRH.

### 3.3. Copy Number Variation Contribute to the Differences in Egg Production by Influencing the Expression Level of AP2M1

**Figure 4:**
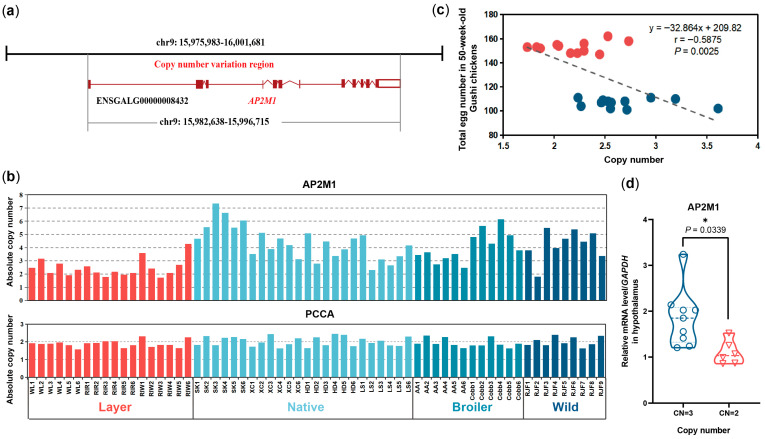
Copy number variation (CNV) in chicken *AP2M1*. (**a**) Gene location of chicken *AP2M1*. (**b**) CNV validation of *AP2M1* gene in different chicken breeds by qRT-PCR. *X*-axis represents all 63 samples from ten breeds. Layer: White Leghorn (WL), Rhode Island Red (RIR), and Rhode Island White (RIW) chickens. Native chickens: Silky (SK), Xichuan black bone (XC), Houdan (HD), Lushi (LS) chickens. Broilers: Arbor Acres broilers (AA) and Cobb broilers (Cobb). Wild breed: Red Jungle Fowl (RJF). The PCCA gene, which was previously identified as a stable, autosomal non-CNV locus with two copies, was used as the control region [15]. (**c**) The correlation between CNV and egg number in Gushi chickens. (**d**) The expression levels of *AP2M1* in hypothalamus of 50-week-old Gushi chickens with different copy numbers (CN = 2 or 3). The data are presented as mean ± SEM. Signifcant diference: * *p* < 0.05.

Chicken *AP2M1* is located in chromosome 9 (chr9), with a total length of 14,077 bp, including 12 exons, 11 introns. According to the CNV identification conducted by Yi et al. (2014) [15], Chicken *AP2M1* (ENSGALG00000008432, GRCg6a:CM000101.5) was included in the copy number variation region (CNVR: chr9:15,975,983–16,001,681) (Figure 4a). We further verified the CNV of *AP2M1* gene in different chicken breeds by qRT-PCR, and identified a copy number loss of *AP2M1* (CN_mean_ = 2.35, CN = 2) in layers compared to the copy numbers in native breeds (CN_mean_ = 4.38, CN = 4), commercial broilers (CN_mean_ = 4.05, CN = 4), and wild breed (CN_mean_ = 4.23, CN = 4) (Figure 4b). The correlation analysis between CNV and egg number in Gushi chickens showed that the CNV was significantly negatively correlated with the total egg number at 50 weeks of age (r = −0.5875, *p* = 0.0025) (Figure 4c). Additionally, the mRNA levels of *AP2M1* in the hypothalamus of Gushi chickens with CN = 2 was significantly lower than that of the individuals with CN = 3 (*p* < 0.05; Figure 4d).

### 3.4. Egg-Laying Related SNP Regulate the Expression Level of AP2M1

**Figure 5:**
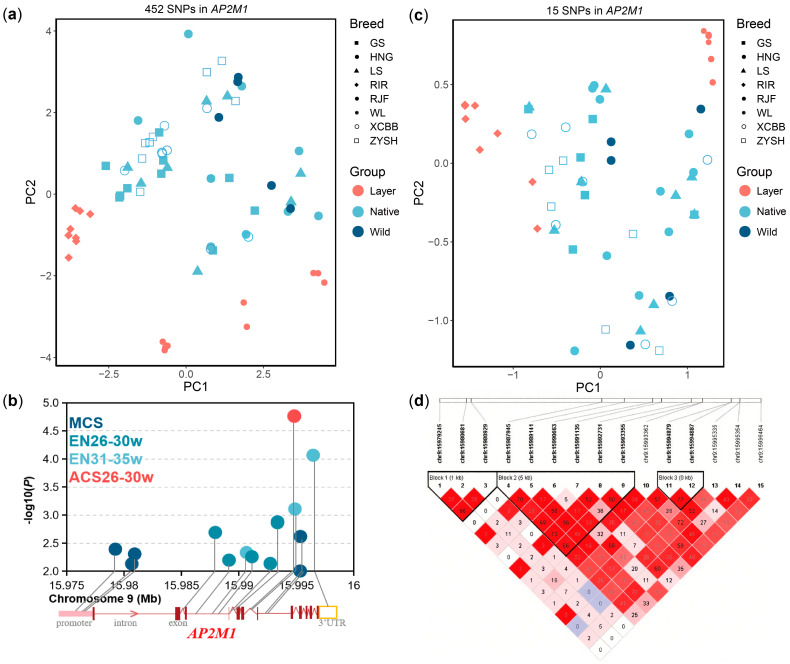
Screening of egg laying-related SNPs mapping to *AP2M1* gene and their population characteristics. (**a**) Principal component analysis (PCA) plots based on SNPs mapping to the 2 kb promoter and gene body of *AP2M1* in multiple breeds. (**b**) Egg laying-related SNPs (*p* < 1 × 10^−2^) on the *AP2M1* gene in Gushi chickens. (**c**) PCA plots based on egg laying-related SNPs in multiple breeds. (**d**) Linkage disequilibrium (r^2^) analysis of egg laying-related SNPs in Gushi chicken population.

Based on whole-genome SNPs in *AP2M1* gene across multiple chicken breeds, we extracted 452 SNPs and observed that layer breeds separated from wild and native breeds via PCA (Figure 5a). We further conducted the association analysis of these SNPs with 13 egg production traits and found that 15 of the SNPs (*p* < 1 × 10^−2^) were significantly associated with the egg number from 26 to 30 weeks of age (EN26-30w), egg number from 31 to 35 weeks of age (EN31-35w), average clutch size from 26 to 30 weeks of age (ACS26-30w), or maximum clutch size (MCS) in Gushi chickens (Figure 5b). The genetic differentiation between layer breeds and wild or native breeds was also evident when we analyzed population structure based on these 15 egg-laying related SNPs of *AP2M1* (Figure 5c). Linkage disequilibrium (LD) analysis of these 15 SNPs in the Gushi chicken population showed that 11 of these SNPs were clustered in three LD blocks with scale of 1684 bp, 5410 bp, and 8 bp, respectively (Figure 5d). Tag SNPs from three LD blocks and the other three SNPs (chr9:15980929G>A in the promoter, chr9:15987945T>A in the intron, chr9:15993362G>A in the intron, chr9:15994879T>C in the intron, chr9:15995354G>A in the intron, chr9:15996464G>A in the 3′UTR) were used for the analysis of the differences in egg production and *AP2M1* expression level among different genotypes.

**Figure 6:**
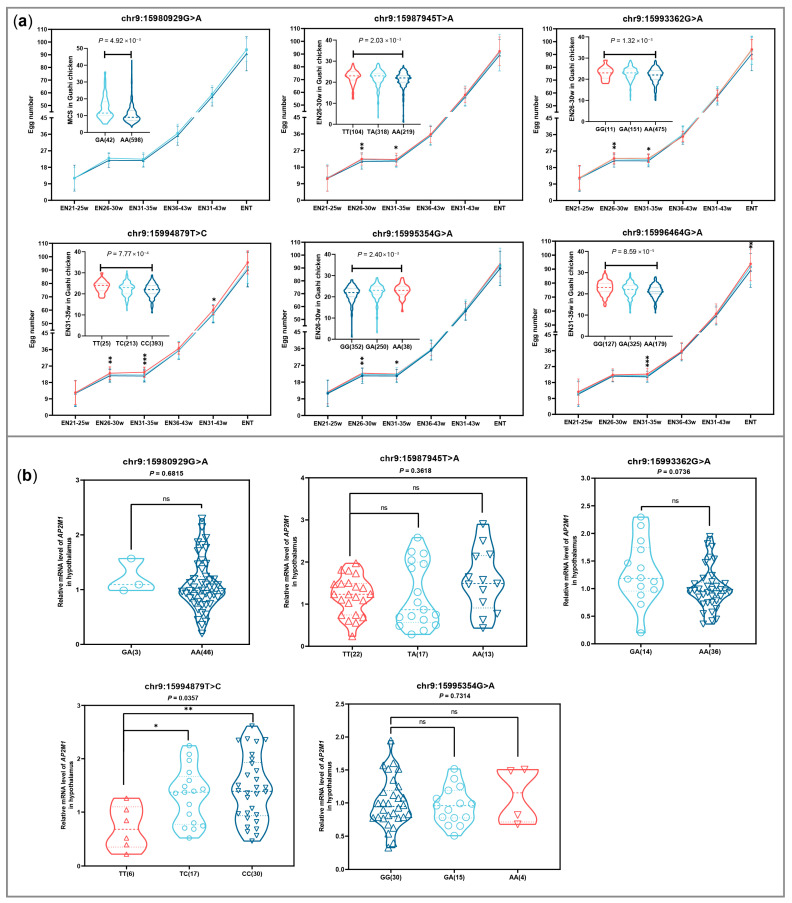
Comparison of egg number (EN) from different egg-laying stages in Gushi chicken population (**a**) and *AP2M1* expression in hypothalamus of 43-week-old Gushi hens (**b**) among the individuals with different genotypes for 6 SNPs including chr9:15980929G>A, chr9:15987945T>A, chr9:15993362G>A, chr9:15994879T>C, chr9:15995354G>A, and chr9:15996464G>A. Each dot represents an individual. “ns” indicates no significant difference. * *p* < 0.05; ** *p* < 0.01; *** *p* < 0.001.

The difference analysis in egg production among the different genotypes of the 6 SNPs showed that 5 SNPs, except for chr9:15980929G>A, can significantly affect egg number at different laying stages (Figure 6a), especially the SNP chr9:15994879T>C for ENT-created phenotypic differences of 5.30 eggs between 2 homozygous genotypes. The difference analysis in expression level among the different genotypes of 5 SNPs except chr9:15996464G>A showed that only chr9:15994879T>C were significantly correlated with *AP2M1* expression levels in chicken hypothalamus tissue (*p* < 0.05; Figure 6b). Additionally, the genotype TT of chr9:15994879T>C was more conducive to increasing egg production, but it is not beneficial for the expression of *AP2M1*, compared to the other genotypes (*p* < 0.05; Figure 6).

**Figure 7:**
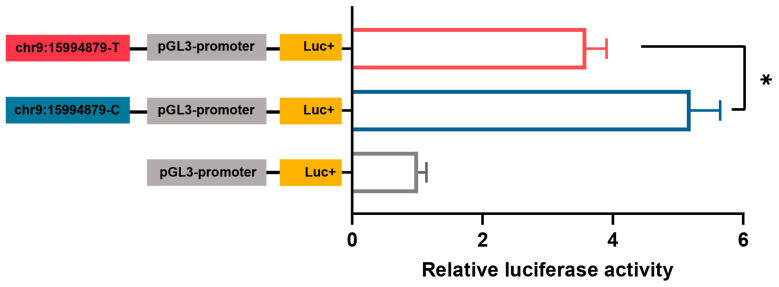
The dual-luciferase assay for the pGL3-promoter containing the genomic region surrounding chr9:15994879-T or chr9:15994879-C in chicken primary hypothalamic neuron cells. The data are presented as mean ± SEM (*n* = 6 for each group). * *p* < 0.05.

Further, we examined the allelic regulatory activity between chr9:15994879T>C and *AP2M1* expression by dual-luciferase reporter assays of the constructed mutant-type chr9:15994879-C and wild-type chr9:15994879-T pGL3-promoter vectors. Result showed that the regulatory activities of mutant-type and wild-type were enhanced, with the chr9:15994879-C allele having significantly enhanced effect compared with the chr9:15994879-T allele (*p* < 0.05; Figure 7).

## 4. Discussion

The genetic regulatory mechanism of egg production trait is complex and driven by multiple genetic variations and candidate genes expressed in multiple tissues [12]. Through the analysis of the tissue expression characteristics and regulatory functions of *AP2M1*, we have verified that *AP2M1* negatively affects egg production in chickens by inhibiting the synthesis and secretion of GnRH in hypothalamic neurons. Additionally, we identified the CNV and the functional SNP chr9:15994879T>C in *AP2M1* that could help account for the differences in egg production among chicken populations by regulating *AP2M1* expression. The identification of these effective genetic molecular markers are critical to translating candidate theory findings into laying-oriented breeding design information.

In poultry, egg laying is a highly coordinated reproductive physiological process that involves neuroendocrine regulatory system, gonadal development, ovulation, and oviposition, and is not only directly controlled by hypothalamic–pituitary–ovarian (HPO) axis tissues, but also indirectly regulated by other peripheral tissues such as liver and abdominal fat [17,21,22]. Due to the widespread expression of *AP2M1* in chickens, we analyzed the differential expression of *AP2M1* in multiple tissues of high- and low-yield chickens. We found that only the expression of *AP2M1* in the hypothalamus was negatively correlated with egg production. Additionally, the negative correlation was observed at different egg-laying stages, in different breeds, and at protein levels, indicating that the target site of *AP2M1* for egg production regulation is in the hypothalamus in chicken. In the HPO axis regulatory system, the hypothalamus, as the center that initiates the HPO axis, synthesizes and secretes gonadotropin-releasing hormone (GnRH), which acts on the anterior pituitary via hypophysioportal system to stimulate gonadotropin cells to synthesize and secrete gonadotropins, namely luteinizing hormone (LHβ) and follicle-stimulating hormone (FSHβ). LHβ and FSHβ are transported to the ovarian tissue through the bloodstream to regulate the development of follicles, the synthesis and secretion of steroid hormones, and ovulation [23]. Based on the negative correlation between *AP2M1* expression and serum FSH, LH and PROG hormone levels as observed in our previous studies [2], as well as the expression characteristics of *AP2M1* in this study, we inferred that *AP2M1* might regulate the changes in serum reproductive hormones by influencing the level of GnRH in the hypothalamus, thereby affecting egg production.

In the hypothalamic nervous system, GnRH hormone plays a decisive role in the initiation and control of reproductive activities [24]. Insufficient or absent secretion of GnRH hormone causes impaired pubertal development and infertility, and exogenous supplementation, however, restores the mice to a relatively normal phenotype [25]. The regulatory mechanism of GnRH hormone production and pulsed-like release is complex. In mammals, “KNDy neurons” composed of kisspeptin neurons, neurokinin B (NKB) neurons, and dynorphin (Dyn) neurons play a key role in regulating the synthesis and secretion of GnRH [26]. NKB and Dyn are upstream of kisspeptin neurons, activating the receptors they express in KNDy neurons and triggering the pulsed-like release of kisspeptin, while kisspeptin can directly act on the receptor GPR54 expressed in GnRH neurons, promoting the release of GnRH [27,28]. In chickens, due to the deletion of kisspeptin and its receptor GPR54 encoding gene during evolution [29], the regulatory mechanism of the chicken GnRH system presents a unique pattern different from that of mammals [30,31]. Our previous research found that tissue factor pathway inhibitor 2 (*TFPI2*), a core gene for egg production derived from the hypothalamus, can partially relieve the inhibitory effect on GnRH secretion by down-regulating the expression level of gonadotropin-inhibiting hormone (*GnIH* or *NPVF*) [17]. In this study, the co-localization relationship between AP2M1 and GnRH was confirmed in the chicken primary hypothalamic neuron cells, and *AP2M1* can inhibit the synthesis and secretion of GnRH. These results suggest that the negative regulation of *AP2M1* may serve as a unique supplementary mechanism governing chicken reproduction. However, as the current research still cannot confirm whether AP2M1 acts directly on GnRH neurons or indirectly via upstream NPVF neurons, the molecular regulatory mechanism by which *AP2M1* exerts this negative regulatory function still requires further in-depth exploration.

Genetic variation is the decisive factor driving the formation of complex traits such as egg production traits. Whole-genome sequencing has revealed that complex traits are shaped by numerous genetic variants (SNPs, Indels, CNVs), through their effects on gene expression, protein function, and cellular signaling pathways [32]. For the identification of genetic variations in egg-laying traits, most studies have mainly focused on SNPs. Numerous potential SNPs associated with chicken egg production traits have been extensively excavated [1,17,33,34]. Additionally, the vast majority of these SNPs were obtained based on the strict suggestive and genome-wide significance thresholds. Still, the largest-effect SNP explains only 5.6% of the phenotypic variance, and all known SNPs collectively account for less than 50% of the heritability for egg production [1], raising the critical question: where is the missing heritability? In fact, most complex traits result from the cumulative effects of dozens to hundreds of small-effect genetic variations [32]. Nevertheless, a large pool of small-effect SNPs related to egg production traits—particularly functional ones in non-coding regions that mainly drive phenotypic differences—remains yet-to-be-identified. The mining of these small-effect SNPs, along with Indels and CNVs, will help explain more of the missing heritability in these traits.

Based on the aforementioned relationship between *AP2M1* expression and egg production as well as GnRH production, we further explored genetic molecular markers that influence *AP2M1* expression from the perspective of genetic regulation. In mammals, both SNP and CNV can lead to the occurrence of neurological disorders in the brain [5,6]. A missense mutation in *AP2M1* impairs CME and causes epileptic and developmental encephalopathy [7], and the CNV of *AP2M1* may contribute to the risk of autism spectrum disorders [35]. In poultry, no functional genetic variations related to *AP2M1* have been reported yet. However, Yi et al. (2014) reported that a gain in CNV including *AP2M1* genomic sequence was found in a broiler breed [15], which provided us with a direction for genetic variation investigation. The CNV region (~25 Kb) encompasses the entire *AP2M1* gene and a portion of the *ABCF3* gene. Since the *ACSF3* gene showed no differential expression in the hypothalamus of egg-laying difference breeds, as well as in the hypothalamic, pituitary, ovarian, liver, abdominal fat, and pectoralis tissues of individual egg-laying difference within the same breed (Figures S1 and S2), our focus was solely on *AP2M1*. Through the verification of CNV containing *AP2M1* genomic sequence in layer breeds, native chicken breeds, and commercial broiler breeds, we found that the copy number of the CNV was the lowest in layer breeds. CNV can affect gene expression levels via containing or disrupting gene coding regions, and *AP2M1* expression characteristics also indicated that its expression in layer breed was significantly lower than that in native chicken breeds, which lead us to speculate that CNV might be related to egg production. Further correlation analysis between CNV and egg number or *AP2M1* expression within the same breed confirmed our speculation, but data from larger populations are still needed to support our conclusion.

In our previous study, by integrating GWAS on egg production traits in GS chickens and multi-tissue transcriptome data from high- and low-yield groups at 43 weeks of age, we identified a series of small-effect SNP sets (*p* < 10^−4^) and 74 core candidate genes, including *AP2M1* gene [17]. In the current study, based on previous resequencing data of multiple breeds and GS chicken population, we extracted all SNP loci in the promoter region and gene body region of *AP2M1*, and screened for small-effect SNPs related to egg production (*p* < 10^−4^) within this region as well as nearby loci within the *p* < 0.01 threshold, aiming to identify small-effect functional variants that may regulate the differential expression of *AP2M1*. Through correlation analysis between egg-laying related SNPs and *AP2M1* expression, we found that among the 15 screened SNP loci, it was not the 2 loci with the strongest correlation—chr9:15994887G>A (*p* = 1.73 × 10^−5^) and chr9:15996464G>A (*p* = 8.59 × 10^−5^)—but rather a neighboring intronic SNP locus, chr9:15994879T>C (*p* = 7.77 × 10^−4^), that influenced *AP2M1* expression. This study has verified the allelic regulatory activity of this locus through luciferase reporter assays. Subsequently, we will continue to delve deeper into the underlying molecular mechanism that the regulation of *AP2M1* transcriptional efficiency by chr9:15994879T>C.

In summary, based on the tissue expression characteristic analysis of *AP2M1*, we verified the negative relationship between *AP2M1* expression and chicken egg production. Overexpression and interference tests in vitro demonstrated that *AP2M1* has a negative regulatory effect on GnRH production in chicken hypothalamic neuron cells. Functional variation mining and preliminary validation indicated that the CNV containing *AP2M1* gene and an intron SNP chr9:15994879T>C may drive the differences in egg-laying phenotype by affecting *AP2M1* expression in chicken. However, the limitations of this work were such that we cannot definitively establish the precise neuronal cellular targets of *AP2M1* in regulating GnRH production, and we are also not clear about the molecular mechanism by which the functional variations regulate *AP2M1* expression.

## 5. Conclusions

In conclusion, we verify that the negative impact of hypothalamic *AP2M1* expression on chicken egg production is achieved by inhibiting the synthesis and secretion of GnRH hormone in hypothalamic neurons. We also identify egg laying-related functional variations including a CNV containing *AP2M1* genomic sequence and an intronic SNP chr9:15994879T>C in *AP2M1*. These functional molecular markers will contribute to the improvement of chicken egg production traits in molecular breeding selection.

## Data Availability

The resequenced raw data from 888 Gushi hens were deposited in the Genome Sequence Archive (GSA) with accession number PRJCA021392. The RNA-seq raw data of 6 tissue types were deposited in NCBI Sequence Read Archive with accession number PRJNA893445 and PRJNA953784. All other data presented in this study are available within the article and its Supplementary Information Files.

## Supplementary Materials

The following supporting information can be downloaded at https://www.mdpi.com/article/10.3390/ani15202990/s1, Table S1: Primer information for luciferase vector construction and qRT-PCR; Table S2: 452 SNPs distributing in 2 kb promoter and gene body of *AP2M1* gene. Figure S1: FPKM of *ABCF3* in different tissues between 43-week-old high- (GS43wH) and low-yield GS chickens (GS43wL). Figure S2: FPKM of hypothalamus *ABCF3* in 30-week-old Rhode Island Red chickens (RIR30w) and Lushi chickens (LS30w).
