# Supplementary material for: Copy Number Variation and SNP Affect Egg Production in Chickens by Regulating AP2M1 Expression to Inhibit GnRH Synthesis
Source: Animals (Basel). 2025 Oct 15;15(20):2990. doi: 10.3390/ani15202990 (PMC12560889; doi:10.3390/ani15202990)
Supplement: Supplementary file 1 [file animals-15-02990-s001.zip › Supplementary Figures.pdf]

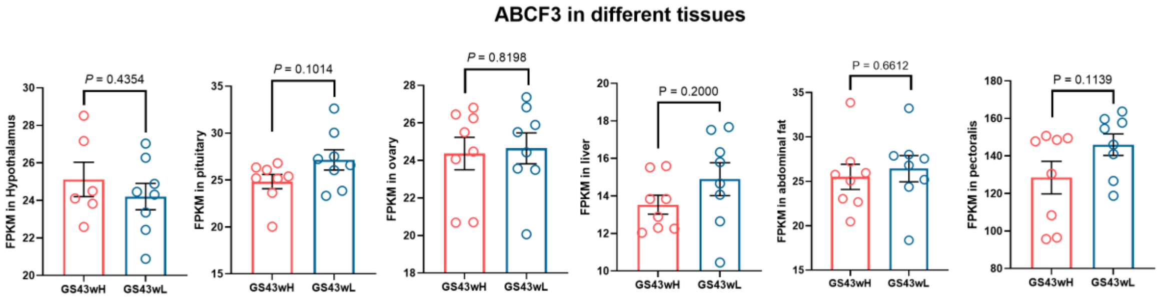

Figure S1. FPKM of *ABCF3* in different tissues between 43-week-old high- (GS43wH) and low-yield GS chickens (GS43wL) (n = 6 for hypothalamus, n =8 for pituitary, ovary, liver, abdominal fat and pectoralis).

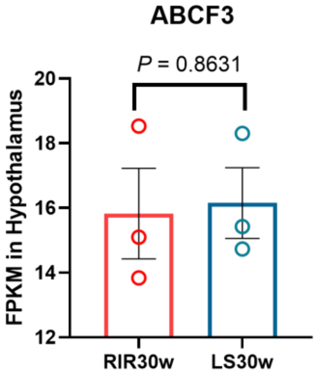

Figure S2. FPKM of hypothalamus *ABCF3* in 30-week-old Rhode Island Red chickens (RIR30w) and Lushi chickens (LS30w) (n = 3).
