# Supplementary material for: Copy Number Variation and SNP Affect Egg Production in Chickens by Regulating AP2M1 Expression to Inhibit GnRH Synthesis
Source: Animals (Basel). 2025 Oct 15;15(20):2990. doi: 10.3390/ani15202990 (PMC12560889; doi:10.3390/ani15202990)
Supplement: Supplementary file 1 [file animals-15-02990-s001.zip › Supplementary Tables.pdf]

Table S1. Primer information for luciferase vector construction and qRT-PCR

Gene	Primer	Sequence(5'-3')	Size (bp)
**A. Primers used for pGL3-promoter vector construction of chr9:15994879T>C**			
AP2M1	Forward	ctagcCCCTGCTGTACTGTCCTC(*NheI*)	201
	Reverse	ctcgagCTCACTAGCCTTGTACTTTG(*XhoI*)	
**B. Primers used for pcDNA3.1-EGFP plasmid vector construction of *AP2M1***			
AP2M1	Forward	ctagcgtttaaacttaagcttATGATCGGAGGCTTATTCATCTATAA(*HindIII*)	1326
	Reverse	gtaccgtcgactgcagaattcCTAGCACCTGGTCTCATAGATCCC(*EcoRI*)	
**C. Specific primers used for qRT-PCR**			
AP2M1	Forward	TGTGTGACGTGATGACTGCT	172
	Reverse	TGATGCTGACTCTTGATGCCT	
GnRH	Forward	TGCTTGGCTCAACACTGGTC	194
	Reverse	CCTTCGATCAGGCTTGCCAT	
NPVF	Forward	CACGCTGCATCTTTTCCGAG	132
	Reverse	CGAGGCACTTTTGCTTCCAC	
GAPDH	Forward	GAACATCATCCCAGCGTCCA	210
	Reverse	CGGCAGGTCAGGTCAACAAC	
**D. Primers used for copy number variation (CNV) validation of *AP2M1* gene by qRT-PCR**			
AP2M1	Forward	GTCTGCTCAGTCTCCATCCG	217
	Reverse	GGAAACAGGGACTCACCTCG	
PCCA	Forward	CAGACACACAGAGCCCATCTCT	65
	Reverse	TGGAGCAGTGGTGGCTGTT	

Table S2. 452 SNPs distributing in 2 kb promoters and gene body of *AP2M1* gene

Chromosome	Position	SNP	REF	ALT	Chromosome	Position	SNP	REF	ALT
9	15973071	9:15973071	A	G	9	15983320	9:15983320	G	A
9	15973077	9:15973077	C	T	9	15983338	9:15983338	G	A
9	15973130	9:15973130	G	A	9	15983397	9:15983397	A	G
9	15973142	9:15973142	C	T	9	15983445	9:15983445	G	C
9	15973215	9:15973215	C	T	9	15983555	9:15983555	A	G
9	15973252	9:15973252	C	T	9	15983574	9:15983574	A	C
9	15973282	9:15973282	T	C	9	15983742	9:15983742	T	A
9	15973306	9:15973306	C	T	9	15983773	9:15983773	T	A
9	15973358	9:15973358	C	T	9	15983888	9:15983888	A	G
9	15973402	9:15973402	G	A	9	15983928	9:15983928	A	G
9	15973465	9:15973465	C	T	9	15983969	9:15983969	T	G
9	15973484	9:15973484	G	T	9	15983977	9:15983977	T	C
9	15973619	9:15973619	C	T	9	15984058	9:15984058	A	G
9	15973627	9:15973627	G	A	9	15984066	9:15984066	G	A
9	15973736	9:15973736	G	A	9	15984314	9:15984314	C	A
9	15973774	9:15973774	C	T	9	15984510	9:15984510	C	T
9	15973784	9:15973784	G	A	9	15984516	9:15984516	C	A
9	15973795	9:15973795	G	T	9	15984567	9:15984567	A	G

9	15973817	9:15973817	G	C	9	15984619	9:15984619	T	C
9	15973855	9:15973855	G	A	9	15984620	9:15984620	G	A
9	15973913	9:15973913	C	T	9	15984639	9:15984639	C	G
9	15973930	9:15973930	T	G	9	15984646	9:15984646	G	A
9	15973953	9:15973953	C	A	9	15984678	9:15984678	A	G
9	15973981	9:15973981	C	T	9	15984704	9:15984704	G	T
9	15974053	9:15974053	C	T	9	15984825	9:15984825	T	C
9	15974105	9:15974105	C	T	9	15984828	9:15984828	G	C
9	15974124	9:15974124	C	T	9	15984899	9:15984899	T	C
9	15974147	9:15974147	T	C	9	15985051	9:15985051	G	A
9	15974154	9:15974154	G	C	9	15985068	9:15985068	T	A
9	15974164	9:15974164	G	A	9	15985071	9:15985071	C	T
9	15974238	9:15974238	A	G	9	15985160	9:15985160	T	A
9	15974319	9:15974319	C	T	9	15985176	9:15985176	G	A
9	15974333	9:15974333	T	C	9	15985227	9:15985227	G	A
9	15974390	9:15974390	T	G	9	15985268	9:15985268	C	T
9	15974407	9:15974407	T	C	9	15985294	9:15985294	T	G
9	15974426	9:15974426	G	A	9	15985319	9:15985319	A	G
9	15974464	9:15974464	G	T	9	15985352	9:15985352	C	T
9	15974469	9:15974469	C	T	9	15985365	9:15985365	G	A
9	15974504	9:15974504	C	T	9	15985503	9:15985503	G	C
9	15974515	9:15974515	C	T	9	15985504	9:15985504	A	T
9	15974532	9:15974532	G	A	9	15985573	9:15985573	C	T
9	15974704	9:15974704	C	T	9	15985574	9:15985574	A	G
9	15974743	9:15974743	C	T	9	15985699	9:15985699	C	T
9	15974745	9:15974745	G	T	9	15985704	9:15985704	C	G
9	15974775	9:15974775	G	A	9	15985783	9:15985783	G	C
9	15974793	9:15974793	T	C	9	15986054	9:15986054	G	A
9	15974827	9:15974827	A	G	9	15986105	9:15986105	A	C
9	15974835	9:15974835	C	T	9	15986255	9:15986255	G	A
9	15974883	9:15974883	C	T	9	15986264	9:15986264	C	G
9	15974898	9:15974898	G	A	9	15986284	9:15986284	G	A
9	15974916	9:15974916	C	G	9	15986310	9:15986310	C	T
9	15974921	9:15974921	T	C	9	15986373	9:15986373	T	C
9	15975128	9:15975128	G	T	9	15986482	9:15986482	T	C
9	15975253	9:15975253	C	T	9	15986488	9:15986488	G	A
9	15975287	9:15975287	C	T	9	15986583	9:15986583	A	G
9	15975334	9:15975334	C	T	9	15986584	9:15986584	C	T
9	15975387	9:15975387	C	A	9	15986632	9:15986632	G	A
9	15975394	9:15975394	T	G	9	15986655	9:15986655	A	G
9	15975500	9:15975500	T	C	9	15986753	9:15986753	A	G
9	15975503	9:15975503	G	C	9	15986790	9:15986790	T	A
9	15975523	9:15975523	C	T	9	15986805	9:15986805	C	T
9	15975526	9:15975526	T	A	9	15987003	9:15987003	G	A

9	15975645	9:15975645	A	G	9	15987006	9:15987006	A	C
9	15975659	9:15975659	G	A	9	15987053	9:15987053	C	T
9	15975685	9:15975685	T	C	9	15987129	9:15987129	T	C
9	15975906	9:15975906	G	A	9	15987271	9:15987271	C	T
9	15975968	9:15975968	G	A	9	15987276	9:15987276	C	G
9	15975975	9:15975975	C	G	9	15987351	9:15987351	G	A
9	15976002	9:15976002	A	G	9	15987364	9:15987364	T	C
9	15976093	9:15976093	T	C	9	15987473	9:15987473	T	C
9	15976168	9:15976168	A	G	9	15987504	9:15987504	C	A
9	15976187	9:15976187	A	G	9	15987529	9:15987529	C	T
9	15976223	9:15976223	A	G	9	15987530	9:15987530	A	G
9	15976259	9:15976259	G	A	9	15987616	9:15987616	T	C
9	15976405	9:15976405	T	G	9	15987941	9:15987941	G	A
9	15976433	9:15976433	T	C	9	15987945	9:15987945	A	T
9	15976436	9:15976436	C	G	9	15988005	9:15988005	A	G
9	15976455	9:15976455	G	A	9	15988211	9:15988211	T	A
9	15976490	9:15976490	C	T	9	15988326	9:15988326	C	T
9	15976534	9:15976534	C	T	9	15988471	9:15988471	A	G
9	15976553	9:15976553	T	C	9	15988514	9:15988514	G	A
9	15976555	9:15976555	A	G	9	15988515	9:15988515	T	C
9	15976571	9:15976571	C	T	9	15988712	9:15988712	T	C
9	15976673	9:15976673	A	G	9	15988714	9:15988714	C	T
9	15976682	9:15976682	C	T	9	15988734	9:15988734	C	T
9	15976756	9:15976756	C	G	9	15988741	9:15988741	C	T
9	15976772	9:15976772	G	A	9	15988806	9:15988806	C	T
9	15976839	9:15976839	G	A	9	15988895	9:15988895	C	T
9	15976850	9:15976850	A	T	9	15989064	9:15989064	C	T
9	15976860	9:15976860	G	T	9	15989074	9:15989074	T	C
9	15976910	9:15976910	C	G	9	15989102	9:15989102	A	G
9	15976947	9:15976947	C	T	9	15989129	9:15989129	C	G
9	15977002	9:15977002	G	A	9	15989141	9:15989141	C	T
9	15977062	9:15977062	C	A	9	15989153	9:15989153	C	T
9	15977077	9:15977077	T	A	9	15989173	9:15989173	A	T
9	15977089	9:15977089	C	T	9	15989229	9:15989229	T	C
9	15977096	9:15977096	T	A	9	15989250	9:15989250	A	C
9	15977130	9:15977130	T	C	9	15989265	9:15989265	A	T
9	15977166	9:15977166	G	A	9	15989300	9:15989300	C	T
9	15977206	9:15977206	T	A	9	15989407	9:15989407	G	A
9	15977233	9:15977233	A	G	9	15989417	9:15989417	G	A
9	15977315	9:15977315	C	T	9	15989588	9:15989588	T	C
9	15977356	9:15977356	A	T	9	15989707	9:15989707	A	G
9	15977418	9:15977418	G	A	9	15989788	9:15989788	C	G
9	15977441	9:15977441	A	G	9	15989829	9:15989829	G	A
9	15977449	9:15977449	C	T	9	15989871	9:15989871	T	A

9	15977562	9:15977562	T	A
9	15977564	9:15977564	G	A
9	15977620	9:15977620	C	T
9	15977727	9:15977727	C	T
9	15977774	9:15977774	G	A
9	15977785	9:15977785	G	A
9	15977822	9:15977822	A	G
9	15977829	9:15977829	A	G
9	15977877	9:15977877	C	T
9	15977909	9:15977909	A	G
9	15977963	9:15977963	A	G
9	15977989	9:15977989	C	T
9	15978053	9:15978053	G	A
9	15978125	9:15978125	C	A
9	15978228	9:15978228	G	A
9	15978290	9:15978290	C	A
9	15978328	9:15978328	C	T
9	15978342	9:15978342	G	T
9	15978454	9:15978454	G	A
9	15978570	9:15978570	A	G
9	15978603	9:15978603	T	C
9	15978663	9:15978663	G	A
9	15978670	9:15978670	C	T
9	15978787	9:15978787	C	G
9	15978789	9:15978789	T	G
9	15978817	9:15978817	T	G
9	15978827	9:15978827	T	C
9	15978886	9:15978886	C	T
9	15979120	9:15979120	C	T
9	15979245	9:15979245	A	G
9	15979279	9:15979279	A	G
9	15979294	9:15979294	A	G
9	15979352	9:15979352	G	A
9	15979359	9:15979359	C	T
9	15979397	9:15979397	G	A
9	15979488	9:15979488	G	A
9	15979578	9:15979578	C	G
9	15979631	9:15979631	C	G
9	15979638	9:15979638	G	A
9	15979671	9:15979671	C	T
9	15979672	9:15979672	G	A
9	15979709	9:15979709	G	T
9	15979751	9:15979751	A	G
9	15979830	9:15979830	G	T
9	15989934	9:15989934	G	A
9	15990024	9:15990024	G	A
9	15990060	9:15990060	C	A
9	15990087	9:15990087	A	G
9	15990120	9:15990120	C	T
9	15990193	9:15990193	A	T
9	15990236	9:15990236	A	G
9	15990279	9:15990279	G	A
9	15990425	9:15990425	C	T
9	15990460	9:15990460	C	T
9	15990461	9:15990461	G	T
9	15990509	9:15990509	G	T
9	15990561	9:15990561	T	C
9	15990576	9:15990576	G	C
9	15990597	9:15990597	T	C
9	15990663	9:15990663	G	A
9	15990800	9:15990800	G	A
9	15990876	9:15990876	G	A
9	15990906	9:15990906	T	A
9	15990949	9:15990949	T	C
9	15991030	9:15991030	T	C
9	15991039	9:15991039	C	T
9	15991071	9:15991071	A	G
9	15991082	9:15991082	C	T
9	15991107	9:15991107	C	T
9	15991135	9:15991135	G	C
9	15991145	9:15991145	A	C
9	15991165	9:15991165	G	A
9	15991180	9:15991180	G	A
9	15991217	9:15991217	A	G
9	15991270	9:15991270	T	C
9	15991274	9:15991274	T	G
9	15991310	9:15991310	A	G
9	15991313	9:15991313	T	G
9	15991537	9:15991537	C	T
9	15991778	9:15991778	G	T
9	15991814	9:15991814	C	T
9	15991833	9:15991833	A	G
9	15991917	9:15991917	T	C
9	15991988	9:15991988	G	A
9	15992009	9:15992009	C	G
9	15992023	9:15992023	A	G
9	15992040	9:15992040	A	G
9	15992048	9:15992048	G	A

9	15979858	9:15979858	A	G	9	15992067	9:15992067	T	C
9	15979944	9:15979944	C	G	9	15992096	9:15992096	G	C
9	15979978	9:15979978	C	T	9	15992104	9:15992104	C	T
9	15980002	9:15980002	G	A	9	15992194	9:15992194	C	T
9	15980076	9:15980076	A	T	9	15992222	9:15992222	A	T
9	15980121	9:15980121	T	A	9	15992228	9:15992228	T	C
9	15980139	9:15980139	C	T	9	15992317	9:15992317	C	T
9	15980150	9:15980150	T	G	9	15992490	9:15992490	T	G
9	15980176	9:15980176	A	G	9	15992542	9:15992542	T	C
9	15980203	9:15980203	G	A	9	15992699	9:15992699	T	G
9	15980238	9:15980238	G	C	9	15992731	9:15992731	A	G
9	15980254	9:15980254	C	A	9	15992788	9:15992788	C	T
9	15980288	9:15980288	A	G	9	15992822	9:15992822	A	C
9	15980289	9:15980289	C	T	9	15992904	9:15992904	T	C
9	15980354	9:15980354	A	G	9	15992926	9:15992926	G	C
9	15980373	9:15980373	T	C	9	15993036	9:15993036	G	A
9	15980391	9:15980391	G	A	9	15993056	9:15993056	G	T
9	15980418	9:15980418	A	G	9	15993072	9:15993072	T	C
9	15980435	9:15980435	G	A	9	15993074	9:15993074	C	T
9	15980462	9:15980462	C	T	9	15993133	9:15993133	A	G
9	15980635	9:15980635	G	T	9	15993151	9:15993151	G	A
9	15980721	9:15980721	A	G	9	15993187	9:15993187	C	T
9	15980777	9:15980777	G	A	9	15993205	9:15993205	C	T
9	15980852	9:15980852	C	T	9	15993287	9:15993287	G	A
9	15980878	9:15980878	T	G	9	15993303	9:15993303	C	T
9	15980929	9:15980929	G	A	9	15993327	9:15993327	C	T
9	15980994	9:15980994	C	T	9	15993355	9:15993355	T	C
9	15980996	9:15980996	C	G	9	15993362	9:15993362	G	A
9	15981031	9:15981031	G	A	9	15993402	9:15993402	C	T
9	15981122	9:15981122	T	C	9	15993410	9:15993410	A	G
9	15981125	9:15981125	A	G	9	15993467	9:15993467	A	G
9	15981148	9:15981148	C	T	9	15993556	9:15993556	G	A
9	15981158	9:15981158	C	A	9	15993606	9:15993606	A	G
9	15981315	9:15981315	G	A	9	15993681	9:15993681	T	C
9	15981415	9:15981415	G	A	9	15993693	9:15993693	C	T
9	15981421	9:15981421	G	A	9	15993734	9:15993734	G	A
9	15981454	9:15981454	A	G	9	15993813	9:15993813	G	A
9	15981491	9:15981491	C	T	9	15993837	9:15993837	A	T
9	15981526	9:15981526	C	T	9	15994105	9:15994105	G	T
9	15981528	9:15981528	C	T	9	15994270	9:15994270	G	C
9	15981665	9:15981665	T	G	9	15994316	9:15994316	T	C
9	15981683	9:15981683	A	G	9	15994334	9:15994334	G	C
9	15981754	9:15981754	C	T	9	15994372	9:15994372	G	C
9	15981776	9:15981776	A	T	9	15994424	9:15994424	G	A

9	15981786	9:15981786	G	A	9	15994455	9:15994455	C	T
9	15981796	9:15981796	C	T	9	15994456	9:15994456	G	A
9	15981847	9:15981847	T	C	9	15994512	9:15994512	C	A
9	15981913	9:15981913	C	G	9	15994524	9:15994524	A	T
9	15981951	9:15981951	G	A	9	15994553	9:15994553	T	C
9	15981972	9:15981972	T	G	9	15994810	9:15994810	G	A
9	15982062	9:15982062	A	G	9	15994821	9:15994821	G	A
9	15982106	9:15982106	A	G	9	15994879	9:15994879	C	T
9	15982207	9:15982207	G	A	9	15994887	9:15994887	A	G
9	15982247	9:15982247	A	G	9	15995028	9:15995028	A	G
9	15982269	9:15982269	G	C	9	15995049	9:15995049	T	G
9	15982282	9:15982282	A	G	9	15995123	9:15995123	C	T
9	15982294	9:15982294	G	C	9	15995129	9:15995129	C	T
9	15982479	9:15982479	C	T	9	15995144	9:15995144	G	C
9	15982503	9:15982503	T	C	9	15995335	9:15995335	C	T
9	15982507	9:15982507	A	G	9	15995349	9:15995349	A	T
9	15982526	9:15982526	C	T	9	15995354	9:15995354	G	A
9	15982571	9:15982571	A	G	9	15995373	9:15995373	G	C
9	15982597	9:15982597	G	A	9	15995402	9:15995402	C	T
9	15982618	9:15982618	C	T	9	15995532	9:15995532	A	G
9	15982762	9:15982762	C	T	9	15995585	9:15995585	T	G
9	15982971	9:15982971	C	T	9	15995600	9:15995600	A	C
9	15982976	9:15982976	C	T	9	15995745	9:15995745	C	T
9	15983096	9:15983096	G	A	9	15995747	9:15995747	G	A
9	15983117	9:15983117	A	G	9	15995783	9:15995783	C	T
9	15983150	9:15983150	C	T	9	15996048	9:15996048	G	T
9	15983173	9:15983173	A	G	9	15996265	9:15996265	G	A
9	15983194	9:15983194	C	T	9	15996273	9:15996273	G	A
9	15983210	9:15983210	C	G	9	15996510	9:15996510	A	G
9	15983291	9:15983291	A	G	9	15996532	9:15996532	G	A
9	15983294	9:15983294	G	A	9	15996549	9:15996549	T	C
9	15983317	9:15983317	A	G	9	15996640	9:15996640	C	T
